# Multiple Introductions and Distinct Genetic Groups of Canada Goldenrod (*Solidago canadensis*) in China Revealed by Genomic Single-Nucleotide Polymorphisms

**DOI:** 10.3390/plants12091734

**Published:** 2023-04-22

**Authors:** Hanyang Lin, Luxi Chen, Junmin Li

**Affiliations:** 1 School of Advanced Study, Taizhou University, Taizhou 318000, China; hylin@tzc.edu.cn; 2 Zhejiang Provincial Key Laboratory of Plant Evolutionary Ecology and Conservation, School of Life Sciences, Taizhou University, Taizhou 318000, China; chenluxi@tzc.edu.cn

**Keywords:** invasive plant, *Solidago canadensis*, genotyping by sequencing, multiple introductions, genetic differentiation, bottleneck

## Abstract

Despite numerous studies reported in the context of ecology, the introduction history of the infamous invasive plant Canada goldenrod (*Solidago canadensis* L.) remains elusive. In the present study, we explored the sources and the number of introduction events of this species from its native areas into China. Using the genotyping-by-sequencing approach, we identified 34,035 selectively neutral single-nucleotide polymorphism (SNP) markers to infer the evolutionary trajectories of 77 *S. canadensis* individuals. Both the principal component analysis and the ADMIXTURE analysis revealed two genetic groups that are sympatric to each other in China and suggested the absence of genetic admixtures. The phylogenetic analysis indicated three feasible introduction routes and multiple introduction events of Canada goldenrod into China. Specifically, the one from the USA directly into China, the other from the USA into China through Japan, and the third from the USA into China through Europe. Based on the site frequency spectrum of these identified SNPs, we inferred strong bottleneck events for both genetic groups, and that the multiple introductions did not rescue the decline of genetic diversity. To conclude, multiple introduction events, genetic bottlenecks, and potential human-mediated spread characterize the introduction history of Canada goldenrod in China. The present study harnesses the power of SNP data in deciphering the evolutionary trajectory of invasive plants and paves the way for future studies concerning the invasion mechanism of Canada goldenrod.

## 1. Introduction

As a distinguishing feature of the Anthropocene, biological invasions pose severe threats to local communities and ecosystems [1,2,3]. For decades, ecologists have proposed an array of hypotheses accounting for invasive success, which could be overarchingly explained by native–alien differences in the context of ecological niche or fitness [4,5,6,7,8,9].

However, there is a genetic paradox that falls out of the scope of direct interactions between invasive and native species. How do alien species persist and subsequently become invasive when they suffer from strong founder effects (bottlenecks) in the meantime [10,11,12,13]? By taking advantage of molecular genetics, we now know that reductions in genetic diversity can possibly be rescued from admixtures, which often result from multiple introductions [14,15,16]. Moreover, whether genetic admixture consistently leads to beneficial consequences on fitness is still under hot debate. Mixed genetic materials may result in higher genetic variances and adapted phenotypes as well as worse survival performance when the habitat difference is subtle between the source and the introduced area [17,18,19,20]. Therefore, a deeper understanding of the putative sources and the demographic history of an invasive species is crucial to decipher its invasive mechanism, which arouses a broad consensus in invasion science but is still limited to specific taxa [21,22,23,24].

Being native to North America, Canada goldenrod (*Solidago canadensis* L.) is now a notorious invasive weed globally [25,26,27]. It is a perennial forb that reproduces both clonally and sexually, imposes strong allelopathic effects on native plants, and shows phenotypic plasticity or local adaptation along environmental gradients, all of which benefit its rapid proliferation and dispersal with or without human-mediated activities [28,29,30,31]. In China, Canada goldenrod initially arrived in Shanghai as an ornamental garden plant in the 1930s [32]. Later, it managed to escape into the wild and is distributed widely in eastern China nowadays [33]. Despite some efforts that have been made, the complexity of introduction events of Canada goldenrod into China needs further examination, the presence of pre- or post-introduction genetic admixture is still elusive, and the demographic history remains completely unknown. Based on a few genomic markers (four AFLP loci), Zhao et al. [34] concluded that Chinese *S. canadensis* possibly originated from multiple native sources and no significant reduction in genetic diversity was found compared to native populations. However, the robustness of these preliminary findings calls for tests owing to the limitation of molecular data available and the lack of samples from other alien origins, such as Europe or neighboring countries of China (such as Japan).

Thanks to the advances in DNA sequencing technologies, it is now possible to sophistically explore the genetic basis underlying invasive success [35]. Specifically, a growing body of studies has harnessed the power of single-nucleotide polymorphisms (SNPs) to reveal the evolutionary imprints of invasive organisms along with their invasion histories [24,36,37]. As an efficient way to identify massive genome-wide SNPs, the genotyping-by-sequencing (GBS) method (also referred to as restriction-site-associated DNA sequencing) is becoming increasingly popular in the fields of ecology and evolution, as well as in invasion science [38,39]. For example, van Boheemen et al. [22] obtained 1022 SNPs from 466 individuals of the common ragweed (*Ambrosia artemisiifolia* L.) species across its native and introduced areas based on the GBS method. They found the presence of multiple introductions of this weed, which may play a critical role in its invasive success. Nevertheless, only a handful of similar cases have been reported, and it is promising to expand the utilization of the GBS approach to the study of Canada goldenrod.

In the present study, we mainly aimed to explore the sources and the number of introduction events of Canada goldenrod from North America into China. We sampled representative individuals of *S. canadensis* from native and introduced areas with a particular emphasis on Chinese ones. We applied a two-enzyme GBS approach to obtain genome-wide SNPs for evolutionary analyses. We first assessed the genetic differentiation and the potential population structure of these individuals. Then, we explored the relationships among native and invasive individuals with phylogenetic analysis. Finally, we inferred the demography of identified genetic groups to determine potential historical population events such as bottlenecks.

## 2. Results

### 2.1. Genetic Clusters Revealed by SNP Data

After the quality control process, the GBS process generated an average of 2.7 Gb of clean data for each accession (Table S1). A total of 34,035 SNPs were identified. The BayeScan analysis did not identify any significant SNP outliers based on the multinomial-Dirichlet model and the allele frequency difference between subpopulations (Figure S1). The *F_ST_* varied from 2.12 × 10^−4^ to 2.30 × 10^−4^ with an average of 2.19 × 10^−4^, and log_10_ (posterior odd) varied from −1.08 to −0.92 with an average of −1.00. Therefore, we kept the 34,035 SNPs for genetic analyses.

**Figure 1:**
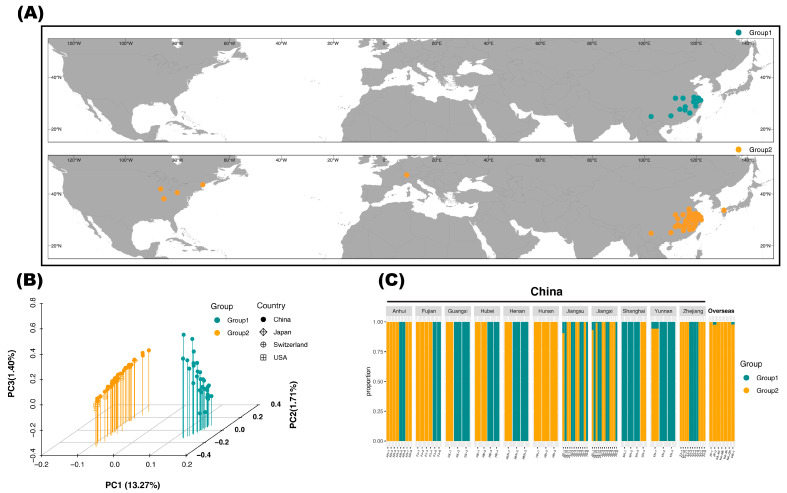
The distribution map and two genetic groups identified among 77 studied *Solidago canadensis* individuals based on 34,035 single-nucleotide polymorphisms (SNPs). (**A**) The distribution map of 77 samples. Labels are colored according to the assigned genetic group consistently throughout the text. Group 1 consisted of 31 Chinese samples. Group 2 comprised 46 individuals from China, the USA, Switzerland, and Japan. (**B**) The distribution of samples along the first three principal components (PC) axes. Group 1 and Group 2 differentiated evidently along PC1, which accounted for 13.27% of the total genetic variances. (**C**) The results of ADMIXTURE analysis showing individual ancestry of 77 samples under the *K* = 2 scenario. The first ancestry was shared by 31 Chinese individuals identical to Group 1, as PCA revealed. The second ancestry was shared by the remaining 46 individuals, which were the same as those comprising Group 2 in the PCA results. The explanation of individual IDs is available from Table S1.

The genetic compositions of 77 studied *S. canadensis* individuals are shown in Figure 1. As indicated by the results of the principal component analysis (PCA), these *S. canadensis* individuals were composed of two distinct genetic groups (Figure 1B). The first group (Group 1 hereafter, with green labels in all figures consistently) and the second group (Group 2 hereafter, with orange labels in all figures consistently) differentiated evidently along the first principal component axis (PC1), which accounted for 13.27% of the total genetic variances. Group 1 consisted of 31 Chinese individuals, while Group 2 consisted of 39 Chinese individuals and 7 overseas samples (2 from Japan, 1 from Switzerland, and 4 from the USA). In China, Group 1 and Group 2 showed a sympatric distribution, i.e., individuals assigned to both genetic groups could be found in the same locality (Figure 1A,B). For example, in Shanghai, where *S. canadensis* was first introduced to China, three of the four studied individuals belonged to Group 1, while the remaining individuals belonged to Group 2. The detailed assignment of genetic group for each individual can be found in Table S1.

Meanwhile, the ADMIXTURE analysis showed congruent results with PCA (Figure 1C). Based on the value of the cross-validation error, *K* = 2 was assumed to be the best model (Table S2). In the *K* = 2 scenario, the 7 out-of-China individuals showed a clear ancestry (assignment of *Q* > 0.95), which was shared by 39 Chinese individuals. These 46 individuals were the same as those comprising Group 2 in the PCA results. Another ancestry was found in 31 Chinese individuals, which were accordingly assigned to Group 1 in the PCA results. Within China, most populations (in the geographical sense) showed a mixture of the two ancestries (except for Hunan; *n* = 3), indicating the absence of a population structure of Chinese *S. canadensis* (Figure 1A,C). The detailed assignment of individual ancestry within each province and the explanation of individual IDs can be found in Figure 1C and Table S1.

### 2.2. Phylogenetic Relationships among Native and Invasive Canada Goldenrod

**Figure 2:**
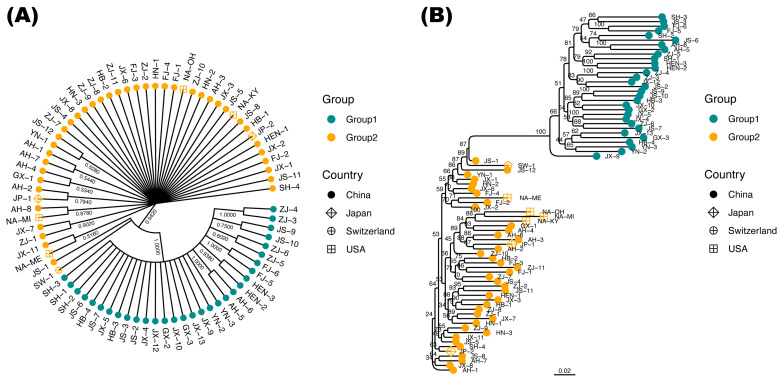
Phylogenetic relationships among 77 *Solidago canadensis* individuals based on 34,035 single-nucleotide polymorphisms (SNPs). (**A**) The consensus neighbor-joining (NJ) tree. The bootstrap support percentage is shown for each node. (**B**) The consensus maximum likelihood (ML) tree. The scale bar is in the unit of the number of substitutions per site. The value of bootstrap support is shown for each node. Both the NJ and the ML tree indicated that individuals from Group 1 were monophyletic (with full bootstrap supports), and the individual showed the closest affinity with Group 1 among those out-of-China samples. The explanation of individual IDs is available in Table S1.

Generally, the phylogeny reconstructed based on two tree inference methods suggested a largely congruent evolutionary history of native and invasive *S. canadensis*. Similar to the results of PCA and ADMIXTURE, sympatric individuals failed to group together in the phylogenetic trees (Figure 2). Both the consensus neighbor-joining (NJ) and the consensus maximum likelihood (ML) trees indicated that individuals from Group 1 were monophyletic (with full bootstrap supports) (Figure 2). The clade comprising Group 1 showed close affinities with one individual from Switzerland and one individual from Jiangsu Province, China (JS-1; sampled from Nantong City).

Given that our sampling scheme was on the individual scale and the internal resolution of the NL tree was relatively poor (extensive polytomies), we put more emphasis on the interpretation of the ML tree. The four USA-derived individuals failed to form a monophyletic clade (Figure 2B). Three samples from Ohio, Michigan, and Kentucky (NA-OH, NA-MI, NA-KY) grouped together, all of which showed a close relationship with two Chinese individuals from Anhui Province (AH-4; sampled from Xuancheng City) and Guangxi Province (GX-1; sampled from Guilin City). However, the remaining USA-derived individual (NA-ME; sampled from Maine) was genetically distant from the other three USA samples and was closest to one Chinese individual collected from Ningde City, Fujian Province (FJ-2; Figure 2B). Similarly, the two Japanese samples were not sister to each other regardless of their identical sampling locality (Fukuoka City, Kyushu). One of the Japanese individuals (JP-1) was sister to one Chinese individual sampled from Hefei City, Anhui Province (AH-2), whereas the other (JP-2) was close to a Shanghai individual (SH-4; Figure 2B). Taken together, the results of the phylogenetic analysis indicate multiple origins and complex invasive routes of Canada goldenrod into China.

### 2.3. Demography of Two Genetic Groups

**Figure 3:**
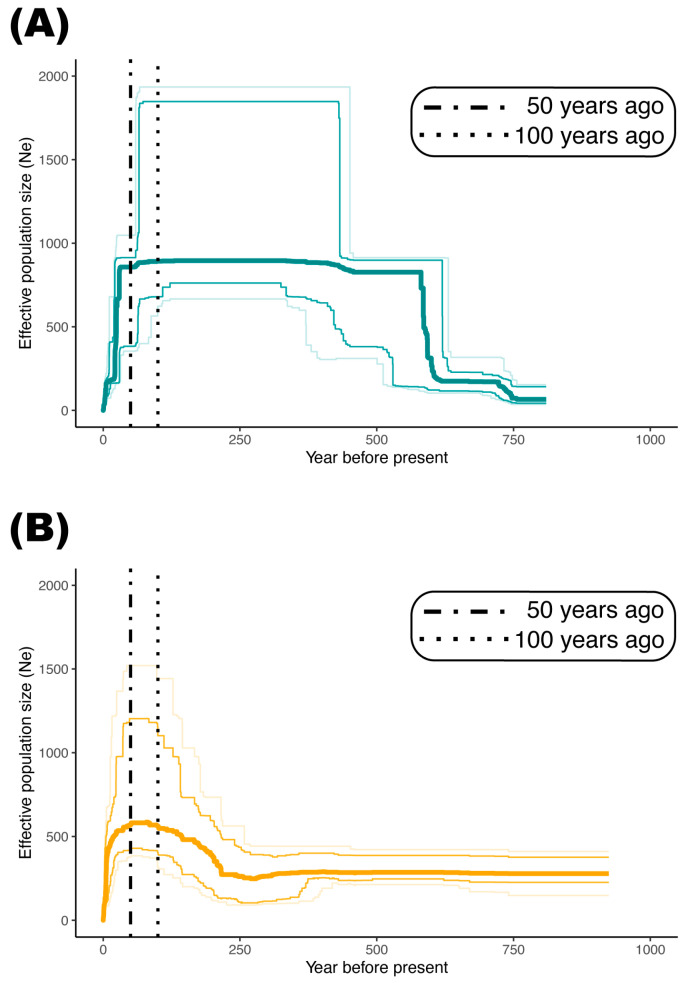
The demographic history of Group 1 (**A**) and Group 2 (**B**) inferred from the folded site frequency spectrum (SFS). The thickest line indicates the median of 100 inferences based on subsampling. The light lines represent 2.5%, 12.5%, 87.5%, and 97.5% estimates of inferences from the top to the bottom, respectively. Group 1 and Group 2 experienced similar demographic histories after their introduction to China (approximately 100 years ago). The two groups have synchronously experienced a severe decline in the effective population size (*N_e_*) within the last 50 years.

The inference of *N_e_* dynamics indicates that Group 1 and Group 2 experienced similar demographic histories after their introduction to China (i.e., approximately 100 years ago) (Figure 3). The two groups have synchronously experienced severe *N_e_* decline within the last 50 years. However, Group 1 and Group 2 differed in *N_e_* trajectories in a long-term view. Group 1 experienced a dramatic population expansion approximately 600 years ago and maintained a relatively high *N_e_* until its introduction to China (Figure 3A). On the contrary, Group 2 maintained a relatively low *N_e_* until a mild increase approximately 200 years ago (Figure 3B).

## 3. Discussion

Similar to what some earlier works have implied, our results clearly reveal multiple introduction events of *S. canadensis* from its native range into China (Figure 2) [27,34]. Not only were the four native USA-derived individuals not monophyletic, but the two Japanese samples failed to group as a clade (Figure 2B). Not surprisingly, one Japanese individual (JP-2) showed the closest affinity with a Shanghai individual (SH-4), which perfectly fits the documented introduction history of *S. canadensis* in China [32]. Therefore, two introduction routes of Canada goldenrod can be safely inferred here: (1) from the USA directly into China; (2) from the USA into China through Japan. Meanwhile, the close phylogenetic relationship between the Switzerland individual and the Group 1 clade, which solely comprises Chinese individuals, implies a third introduction route from the USA into China through Europe (Figure 2B). According to the literature, all three proposed introduction routes are feasible for *S. canadensis* [26,27,40]. Nevertheless, the present work appears to be the first study to confirm the dispersal of Canada goldenrod from Europe into China based on genetic lines of evidence. Moreover, at least five independent introduction events were identified through these routes (Figure 2B). In a nutshell, despite some limitations regarding the number of studied individuals, our novel findings provide new insights into the complicated invasion history of *S. canadensis*.

Largely congruent with previous studies, we found no significant population structure of invasive *S. canadensis* (Figure 1 and Table S2) [41,42]. Meanwhile, we found that most sympatric (i.e., distributed at the same locality) individuals belonged to different genetic groups in China (Figure 1). Long-range dispersal by wind and the occurrence of human- or animal-mediated spread may partly explain this genetic pattern, which is also suggested by the phylogenetic analysis here (Figure 2) [43]. It is well acknowledged that people facilitate biological invasion by altering local ecosystems and also by carrying invasive species to novel localities [44]. Together with our results, we claim that the invasion success of *S. canadensis* in China benefits from the help of human activities and its competitive nature [28]. Recently, *S. canadensis* has been added to the updated list of 33 key invasive alien species “http://www.moa.gov.cn/govpublic/KJJYS/202211/t20221109_6415160.htm” (accessed on 1 March 2023), showing that its management and control remain urgent in China. Hence, the importance of prevention and restoration actions in disturbed habitats where Canada goldenrod has invaded cannot be overstated [2]. Manual cleaning and chemical control have proved to be time-consuming and inefficient [45,46]. Nevertheless, biological control approaches have emerged as an effective way to limit the growth of *S. canadensis* [28]. Some dodder species (*Cuscuta* spp., Convolvulaceae) can parasitize *S. canadensis* and then significantly decrease the growth rate, disturb the mating behaviors, and inhibit the accumulation of biomass of *S. canadensis* [47,48]. Specifically, the parasitic roots of *Cuscuta japonica* are able to parasitize the stems and leaves of *S. canadensis* by penetrating the stem and leaf epidermis to the pith of Canada goldenrod, taking a large amount of water and nutrition from the host, while the parasitism of *Cuscuta australis* could inhibit the growth of *S. canadensis* by altering the resource allocation patterns as well as reducing the resources obtained by the host. Further expedition and utilization of native dodder biological resources may be promising for controlling *S. canadensis* in China.

Earlier genetic analyses suggested somehow contrasting genetic consequences of *S. canadensis* on introduction to different areas. Alexander et al. [49] found that genetic variation within and across populations of *S. canadensis* was significantly reduced in Europe (Canton Valais, Switzerland), suggesting a strong genetic bottleneck, while Zhao et al. [34] found similar genetic variation between fifteen North American and thirteen Chinese populations. However, we argue that these results may be biased and only reflect a small and random fraction of genetic variance due to the limits of AFLP data [50]. Based on the site frequency spectrum of massive genome-wide SNPs, we identified an evident bottleneck event for both genetic groups, which perfectly fits the introduction event and invasion history of *S. canadensis* in China chronologically (Figure 3) [32,33]. Contradicting the prediction by Uller and Leimu [16], multiple introduction events failed to generate genetic admixtures (indicated by the clear individual ancestry; Figure 2) and rescue the reduced genetic diversity (as proxied by *N_e_*) of Chinese *S. canadensis* subsequently (Figure 3). The type of breeding system may partly account for this. Another famous invasive plant, *Ambrosia artemisiifolia* L. (common ragweed, Asteraceae), whose global invasion success was largely facilitated by the pre-introduction admixtures in its native ranges, shows a complicated mating system including selfing, biparental inbreeding, and outcrossing [22,51,52]. Given that *S. canadensis* is highly self-incompatible, the absence of mixed genotypes highlights the important role of clonal reproduction in the survival and spread of *S. canadensis*, as observed in field experiments [27,28,43]. As for the relatively higher *N_e_* (which can be translated into relatively higher genetic diversity) observed in Group 1 when the founder effect began, one possible explanation could be the presence of genetic adaptation during its colonization in Europe, as reported recently by Eckert et al. [42].

Similar to many poster-child invasive plants (e.g., *Reynoutria japonica* Houtt., the Japanese knotweed; *Taraxacum officinale* G. H. Weber ex Wiggers, the dandelion), the low genetic diversity together with the phenotypic plasticity of *S. canadensis* in China strongly implies that epigenetic variations could play critical roles in its invasion success [30,53,54]. With the rapid proliferation of epigenetic, transcriptomic, and genomic data, we are now able to gain some insights into the molecular mechanisms underlying epigenetic variations in detail [55,56]. Among these, DNA methylation (modification of DNA sequences through the addition or removal of methyl groups at cytosine bases), chromatin remodeling through histone modifications, and gene regulations by non-coding RNAs are three of the most well-known processes, which can interactively influence gene expressions and subsequently lead to phenotypic variations [57]. For example, Verhoeven et al. studied a single clone of the invasive dandelion to investigate how exposure to various types of stress (including low nutrients, low salinity, herbivory, and pathogens) would impact DNA methylation across its genome [53]. They found that genome-wide DNA methylation patterns were altered under all stress conditions, and they also found these changes were most evident in clones that were exposed to biotic stress conditions (i.e., herbivory and pathogens). Moreover, most of these epigenetic changes were heritable and persisted in second-generation plants grown under common garden conditions [53]. Compared to DNA methylation, the role of histone modifications in phenotypic variation during biological invasions is less understood. Nevertheless, strong effects of histone modifications on the interactions between fungi and their host plants have been documented, as reviewed by Jeon [58]. On the other hand, it is now well appreciated that non-coding RNAs are of great evolutionary importance, serving as a hidden layer of internal signaling that modulates gene expressions [59,60]. Despite their important roles in gene regulations, a handful of case studies have been reported that directly tested the role of non-coding RNAs in phenotypic responses in invasive plants. For example, Qin et al. investigated whether the differences in gene expressions in response to salinity stress in invasive smooth cordgrass (*Sporobolus alterniflorus* (Loisel.) P.M.Peterson and Saarela) were related to non-coding RNAs’ activities [61]. They found that non-coding RNAs were more active under stress conditions, providing insights into the potential involvement of non-coding RNAs in mediating salt tolerance in this notorious invasive plant [61]. By comparing gene and microRNA (miRNA; a type of highly conserved non-coding RNA molecule) expression profiles between two levels of ploidy (diploid vs. hexaploidy), Xu et al. found that both gene and miRNA regulators contributed to the successful invasion of hexaploid *S. canadensis* [62]. A great number of differentially expressed genes were significantly associated with the biosynthesis of secondary metabolites, carbohydrate metabolism, lipid metabolism, and environmental adaptation pathways. Meanwhile, miRNA–gene interaction networks have been determined to strongly influence the expressions of genes encoding transcription factors, DNA methyltransferase, and leucine-rich repeat receptor-like kinases involved in stress responses. The authors also inferred that the ploidy-related regulation of DNA methylation could serve as an additional modulatory mechanism to drive invasion success [62]. Still, we are desperately hungry for more empirical studies that focus on potential causal relationships between the epigenetic variation and the invasion success of *S. canadensis*. Therefore, we strongly recommend in-depth explorations of epigenetic variations and their underlying molecular mechanisms in future works that aim at decoding the invasion mechanisms of Canada goldenrod in China.

## 4. Materials and Methods

### 4.1. Sample Collection

We sampled a total of 77 *S. canadensis* individuals during 2012–2016, including 70 individuals from China, 2 from Japan, 1 from Switzerland, and 4 from the USA (Figure 1). The detailed information is shown in Table S1. Among the Chinese samples, 8 were collected from Anhui Province (six cities), 6 were from Fujian Province (four cities), 3 were from Guangxi Province (Guilin City), 3 were from Henan Province (Xinyang City), 4 were from Hubei Province (two cities), 3 were from Hunan Province (three cities), 12 were from Jiangsu Province (six cities), 13 were from Jiangxi Province (seven cities), 3 were from Yunnan Province (Kunming City), 11 were from Zhejiang Province (seven cities), and 4 were from Shanghai City, largely representing the current distribution of invasive Canada goldenrod in China (Figure 1A; Table S1). Given that no previous genetic study has included *S. canadensis* from possibly intermediate invasion sites (such as Europe and Japan) to explore the invasion routes of this species in China, we collected one European (Elfingen, Aargau, Switzerland) and two Japanese (Fukuoka, Kyushu) samples as representative individuals (Table S1). The four native samples were collected from the center area of the USA to the northeastern states (Simpsonville, Kentucky; Portland, Maine; Stevensville, Michigan; and Salem, Ohio, respectively), where *S. canadensis* can be readily found. Caveats we took into consideration during the sampling process in China were fully explained by Wan et al. [63]. Generally, we sampled *S. canadensis* individuals that occurred on loose and dry soils to avoid accidental collections of its congeners.

### 4.2. GBS and SNP Calling

The GBS procedure was conducted following the original protocol [64,65]. First, the genomic DNA of silica-gel-dried materials was isolated using the Plant DNAzol™ Reagent (Invitrogen, Carlsbad, CA, USA). Then, the DNA was digested with the *Eco*RI and *Nla*III enzymes, and the digested products were mixed with adapters (New England Biolabs, Ipswich, MA, USA). Later, the DNA libraries were pooled, size-selected (300–400 bp) on 1% agarose gels, column-cleaned using PCR purification kits, and amplified for 12 cycles using Phusion™ DNA polymerase (New England Biolabs, Ipswich, MA, USA). The average fragment size was estimated on a 2100 Bioanalyzer Instrument (Agilent, Santa Clara, CA, USA), and the library quantification was performed using the PicoGreen™ kit (Invitrogen, Carlsbad, CA, USA). Finally, the pooled libraries were sequenced with the NovaSeq™ 6000 platform to generate paired-end reads of 150 bp (Illumina, San Diego, CA, USA). All the sequencing data have been deposited at GenBank under the accession number PRJNA937432.

We conducted the SNP calling with the following steps. First, the raw sequencing data were processed using fastp (version 0.18.0) [66] to remove the adapter sequences and low-quality sequences (reads with ≥10% unidentified nucleotides (Ns) or with >50% bases having Phred quality scores of ≤20). Then, the *de novo* assembly of clean reads and the clustering of tags were performed using the Stacks (version 1.43) pipeline [67]. Basically, clean reads of each sample were assembled if they overlapped. The non-overlapping and overlapping reads were clustered and merged into a non-redundant assembled reference genome. Clean reads of each sample were mapped to the assembled reference genome using the Burrows–Wheeler Alignment (BWA) tool (version 0.7.12) [68]. Next, we performed variant calling using the Genome Analysis Toolkit (GATK) Best Practices Pipeline (version 3.4) [69]. Later, identified variants were hard-filtered with the VariantFiltration tool from GATK (-Window 4, -filter “QD < 2.0 || FS > 60.0 || MQ < 40.0”, -G_filter “GQ < 20”) and soft-filtered using VCFtools (version 0.1.13) (--remove-indels --max-missing 0.8 --maf 0.05) [70].

### 4.3. Analysis of Population Structure

Prior to the subsequent analyses, we tested whether these retained SNPs were selectively neutral using BayeScan (version 2.1) with the default settings [71]. Based on the putatively neutral SNPs, we determined genetic clusters within the 77 *S. canadensis* individuals with PCA using PLINK (version 1.9) [72]. Meanwhile, we estimated the individual ancestries using ADMIXTURE (version 1.3.0) [73]. Five bootstrap replicates were performed, each testing for two to ten clusters (*K*) with 5-fold cross-validations.

### 4.4. Reconstruction of Phylogeny

To explore the evolutionary affinities among *S. canadensis* individuals with different geographical origins, we reconstructed the individual phylogeny based on the putatively neutral SNPs with both the NJ method and the ML method [74]. The consensus NJ tree was inferred using MEGA (version 11.0.11) with 500 bootstrap replicates [75,76]. The consensus ML tree was inferred using IQ-TREE (version 2.2.0.3) with 1000 ultra-fast bootstrap replicates [77,78]. As implemented in IQ-TREE, the DNA substitution model was inferred with ModelFinder [79]. Both the NJ and ML tree were un-rooted.

### 4.5. Demography Inference

We inferred the demographic history of the two distinct genetic groups of *S. canadensis* (see Results) as follows. First, we obtained the folded site frequency spectrum (SFS) using the SNPs identified above and easySFS “https://github.com/isaacovercast/easySFS” (accessed on 4 September 2022). Then, the effective population size (*N_e_*) of the two genetic groups over time was simulated using Stairway Plot 2 (version 2.1.1) [80]. The generation time of *S. canadensis* was assumed to be one year [27]. The mutation rate was set to 7 × 10^−9^ base substitutions per site per generation following *Arabidopsis thaliana* [81]. For each group, the *N_e_* dynamics were inferred with 100 estimates.

## 5. Conclusions

By exploring genomic SNPs of both the native and invasive Canada goldenrod individuals, our results indicate that multiple introduction events, genetic bottlenecks, potential human-mediated dispersal events, and clonal reproduction all play roles in the invasion history of Canada goldenrod into China. The present study highlights the power of genome-wide SNPs in inferring the evolutionary history of invasive plants and paves the way for future studies regarding the invasion mechanism of *S. canadensis*.

## Data Availability

The data presented in this study are openly available in GenBank under the accession number PRJNA937432.

## Supplementary Materials

The following supporting information can be downloaded at: https://www.mdpi.com/article/10.3390/plants12091734/s1. Figure S1: the BayeScan analysis was based on the multinomial Dirichlet model and the allele frequency difference between subpopulations. The red vertical line represents a zero value of log_10_(posterior odd); Table S1: information of 77 sequenced *Solidago canadensis* individuals; Table S2: cross-validation errors in ADMIXTURE analysis.
