# Supplementary material for: Multiple Introductions and Distinct Genetic Groups of Canada Goldenrod (Solidago canadensis) in China Revealed by Genomic Single-Nucleotide Polymorphisms
Source: Plants (Basel). 2023 Apr 22;12(9):1734. doi: 10.3390/plants12091734 (PMC10180931; doi:10.3390/plants12091734)
Supplement: Supplementary file 1 [file plants-12-01734-s001.zip › plants-2342862-supplementary.pdf]

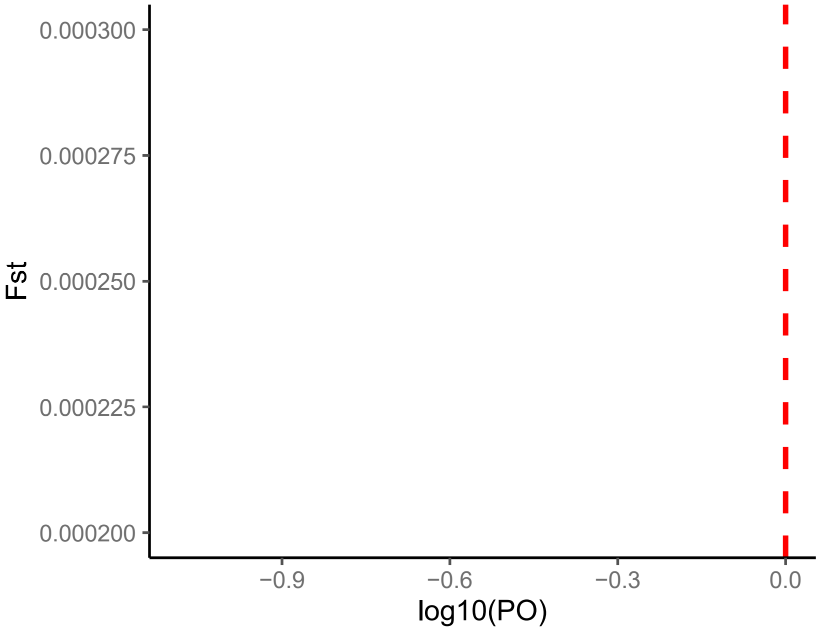

Figure S1. The result of BayeScan analysis based on the multinomial-Dirichlet model and the allele frequency difference between subpopulations. The red vertical line represents a zero value of log10(posterior odd)

Table S1. Information of 77 sequenced *Solidago canadensis* individuals

Country	Province/State	City	Longitutde	Latidude	ID	Data size (Gb)	Genetic group
China	Anhui	Chuzhou	118.4022	32.1967	AH-1	2.615	Group2
China	Anhui	Hefei	117.3792	31.7278	AH-2	2.751	Group2
China	Anhui	Anqing	116.9486	30.7119	AH-3	1.431	Group2
China	Anhui	Xuancheng	118.9739	30.6125	AH-4	1.890	Group2
China	Anhui	Xuancheng	118.9739	30.6125	AH-5	3.762	Group1
China	Anhui	Xuancheng	118.9739	30.6125	AH-6	3.468	Group1
China	Anhui	Huangshan	118.2850	29.7281	AH-7	2.957	Group2
China	Anhui	Bengbu	117.4047	32.9364	AH-8	3.806	Group2
China	Fujian	Wuyishan	118.0100	27.2300	FJ-1	1.247	Group2
China	Fujian	Ningde	118.7400	26.5600	FJ-2	1.929	Group2
China	Fujian	Nanping	118.9800	27.1800	FJ-3	1.376	Group2
China	Fujian	Sanming	117.5600	26.2400	FJ-4	1.941	Group2
China	Fujian	Sanming	117.5600	26.2400	FJ-5	3.560	Group1
China	Fujian	Sanming	117.5600	26.2400	FJ-6	3.989	Group1
China	Guangxi	Guilin	110.2881	25.1567	GX-1	1.772	Group2
China	Guangxi	Guilin	110.2881	25.1567	GX-2	3.383	Group1
China	Guangxi	Guilin	110.2881	25.1567	GX-3	3.072	Group1
China	Henan	Xinyang	114.9156	32.0008	HEN-1	2.062	Group2

China	Henan	Xinyang	114.9156	32.0008	HEN-2	3.660	Group1
China	Henan	Xinyang	114.9156	32.0008	HEN-3	3.554	Group1
China	Hubei	Qianjiang	112.9363	30.3943	HB-1	2.308	Group2
China	Hubei	Xiangyang	112.0391	31.9937	HB-2	1.917	Group2
China	Hubei	Xiangyang	112.0391	31.9937	HB-3	3.482	Group1
China	Hubei	Xiangyang	112.0391	31.9937	HB-4	3.428	Group1
China	Hunan	Changsha	112.9500	28.1400	HN-1	2.112	Group2
China	Hunan	Loudi	111.9800	27.4200	HN-2	3.018	Group2
China	Hunan	Xiangtan	112.8800	27.8500	HN-3	1.540	Group2
China	Jiangsu	Nantong	120.8422	32.0715	JS-1	3.735	Group2
China	Jiangsu	Nantong	120.8422	32.0715	JS-2	2.660	Group1
China	Jiangsu	Nantong	120.8422	32.0715	JS-3	2.966	Group1
China	Jiangsu	Xuzhou	117.2953	34.1972	JS-4	1.668	Group2
China	Jiangsu	Changzhou	119.9378	31.8781	JS-5	3.680	Group2
China	Jiangsu	Changzhou	119.9378	31.8781	JS-6	3.382	Group1
China	Jiangsu	Changzhou	119.9378	31.8781	JS-7	4.091	Group1
China	Jiangsu	Yangzhou	119.1586	32.3772	JS-8	2.306	Group2
China	Jiangsu	Yangzhou	119.1586	32.3772	JS-9	3.860	Group1
China	Jiangsu	Yangzhou	119.1586	32.3772	JS-10	3.878	Group1
China	Jiangsu	Taizhou	119.9742	32.5289	JS-11	0.898	Group2
China	Jiangsu	Suzhou	120.5828	31.3361	JS-12	0.821	Group2
China	Jiangxi	Jiujiang	116.0327	29.7330	JX-1	1.932	Group2
China	Jiangxi	Yingtan	117.0531	28.2719	JX-2	2.896	Group2
China	Jiangxi	Nanchang	115.8136	28.6636	JX-3	1.915	Group2
China	Jiangxi	Nanchang	115.8136	28.6636	JX-4	4.015	Group1
China	Jiangxi	Nanchang	115.8136	28.6636	JX-5	3.527	Group1
China	Jiangxi	Ji’an	115.2980	27.5950	JX-6	2.462	Group2
China	Jiangxi	Ganzhou	115.0589	25.9286	JX-7	1.359	Group2
China	Jiangxi	Pingxiang	113.7636	27.6466	JX-8	3.392	Group2
China	Jiangxi	Pingxiang	113.7636	27.6466	JX-9	4.315	Group1
China	Jiangxi	Pingxiang	113.7636	27.6466	JX-10	3.975	Group1
China	Jiangxi	Fuzhou	115.7340	27.3843	JX-11	3.427	Group2
China	Jiangxi	Fuzhou	115.7340	27.3843	JX-12	3.677	Group1
China	Jiangxi	Fuzhou	115.7340	27.3843	JX-13	3.352	Group1
China	Yunnan	Kunming	102.6500	24.9100	YN-1	3.429	Group2
China	Yunnan	Kunming	102.6500	24.9100	YN-2	3.398	Group1
China	Yunnan	Kunming	102.6500	24.9100	YN-3	3.307	Group1
China	Zhejiang	Ningbo	121.1762	30.3600	ZJ-1	2.299	Group2
China	Zhejiang	Jinhua	119.8701	28.9300	ZJ-2	1.183	Group2
China	Zhejiang	Jinhua	119.8701	28.9300	ZJ-3	3.533	Group1

China	Zhejiang	Jinhua	119.8701	28.9300	ZJ-4	3.852	Group1
China	Zhejiang	Huzhou	120.3993	30.7400	ZJ-5	3.862	Group1
China	Zhejiang	Huzhou	120.3993	30.7400	ZJ-6	3.901	Group1
China	Zhejiang	Huzhou	120.3993	30.7400	ZJ-7	1.877	Group2
China	Zhejiang	Zhoushan	121.9980	30.0500	ZJ-8	1.880	Group2
China	Zhejiang	Jiaxing	121.0270	30.6000	ZJ-9	2.368	Group2
China	Zhejiang	Shaoxing	120.5164	30.1500	ZJ-10	3.764	Group2
China	Zhejiang	Lishui	119.5945	28.0978	ZJ-11	1.180	Group2
China	-	Shanghai	121.3080	31.2064	SH-1	2.746	Group1
China	-	Shanghai	121.3080	31.2064	SH-2	2.842	Group1
China	-	Shanghai	121.8002	31.1622	SH-3	3.042	Group1
China	-	Shanghai	121.8002	31.1622	SH-4	1.985	Group2
Japan	Kyushu	Fukuoka	130.7121	33.7835	JP-1	1.714	Group2
Japan	Kyushu	Fukuoka	130.7406	33.7270	JP-2	2.744	Group2
Switzerland	Aargau	Elfingen	8.3477	47.4470	SW-1	2.271	Group2
USA	Kentucky	Simpsonville	-85.3300	38.2036	NA-KY	1.049	Group2
USA	Maine	Portland	-70.3186	43.6533	NA-ME	1.374	Group2
USA	Michigan	Stevensville	-86.5175	42.0314	NA-MI	0.857	Group2
USA	Ohio	Salem	-80.7570	40.5980	NA-OH	0.979	Group2

Table S2. Cross-validation errors in ADMIXTURE analysis

K	Cross-validation error
2	0.4289
3	0.4531
4	0.4952
5	0.5340
6	0.5978
7	0.6304
8	0.7052
9	0.7625
10	0.8183
